# Prescription Department

**Published:** 1898-02

**Authors:** 


FOR INFLUENZA.

℞. Quin. sulph............. XLV gr.
Pulv. digitalis..................
Pulv. scillæ...........aa gr. xv.
Ext. opii....................gr. v.
Ext. glycyrrhizæ...........q. s.
M. ft. pil. No. xxx. Sig. Four pills daily.—*Pepper, Ex.*

VOMITING FOLLOWING IN THE COURSE OF INTESTINAL AFFECTIONS.

℞. Menthol................0.50 cgm.
Cognac...............40 gm.
Simple tincture of opium............10 gm.
M. Sig. Take twenty drops several times daily.—*Peck*, *Journal de Medi·ine de Paris*, June 27, 1897.

FOR REMOVAL OF WARTS AND CORNS.

℞. Acid salicylic............2 ʒss.
Ext. cannabis indic.....33 gr. v.
Collodion flex...........16 ℥ss.
Sig. To be painted over such an area, twice daily, for three days, when an oil poultice is applied over night —*Medical and Surgical Reporter.*

CHRONIC CYSTITIS.

℞. Fluid extract golden-seal.. ʒ ij

Tincture gentian comp..... ʒ iv.

Tincture staphisagria....... ʒ i.

Tincture cannabis indica..... ʒ i.

Syrup bitter orange peel.... ℥ iv.

A teaspoonful three times daily.—*Medical Fortnightly.*

